# Esophageal Involvement in Non–Eosinophilic Esophagitis Eosinophilic Gastrointestinal Diseases: Association With Esophageal Symptoms and Gastric Involvement

**DOI:** 10.1002/jgh3.70449

**Published:** 2026-07-22

**Authors:** Yasuhiko Abe, Naoko Mizumoto, Yu Sasaki, Makoto Yagi, Yusuke Onozato, Yoshiyuki Ueno

**Affiliations:** ^1^ Division of Endoscopy Yamagata University Hospital Yamagata Japan; ^2^ Department of Gastroenterology, Faculty of Medicine Yamagata University Yamagata Japan

**Keywords:** endoscopy, eosinophilic gastritis, eosinophilic gastrointestinal disease, EREFS, esophageal involvement

## 1 Introduction

Eosinophilic gastrointestinal diseases (EGIDs) are chronic inflammatory disorders characterized by a T helper type 2 (Th2)–predominant immune response, eosinophilic infiltration of the gastrointestinal tract, and related gastrointestinal symptoms [1]. EGIDs are broadly classified into eosinophilic esophagitis (EoE), which is confined to the esophagus, and non–EoE EGIDs, which can affect other segments of the gastrointestinal tract, including the stomach, small intestine, and colon.

Non–EoE EGIDs often involve multiple gastrointestinal segments through mechanisms that remain incompletely understood. Accordingly, site‐based classifications—such as eosinophilic gastritis (EoG), eosinophilic duodenitis (EoD), eosinophilic enteritis (EoN), and eosinophilic colitis (EoC)—have been proposed to better characterize the pathophysiology of disease [2].

Recent studies have reported that approximately 20%–40% of patients with non–EoE EGIDs exhibit esophageal involvement (EI) [3, 4, 5]. Moreover, EI has been suggested to be associated with a greater disease burden, including more complex diagnoses, treatments, and prognoses [4, 6]. However, the clinical significance and characteristics of EI in patients with non‐EoE EGID remain unclear.

Therefore, this study aimed to evaluate the clinical and endoscopic characteristics associated with EI and to examine their relationship with involvement at other gastrointestinal sites in patients with non–EoE EGIDs.

## 2 Methods

We conducted a secondary analysis of patients from a previously reported multicenter study of gastric endoscopic findings in non–EoE EGIDs [7]. The original cohort consisted of 64 patients diagnosed with non–EoE EGIDs according to the Japanese diagnostic criteria. Esophageal biopsies were performed in 29 patients. One patient was excluded because the esophageal eosinophil count was unavailable. Among the remaining 28 patients, those who fulfilled the Pesek histologic criteria for non–EoE EGIDs (stomach ≥ 30 eosinophils per high‐power field [eos/HPF]; small intestine ≥ 50 eos/HPF; colon ≥ 60 eos/HPF) [3] were included. A total of 22 patients met these criteria at one or more gastrointestinal sites and constituted the study population.

Biopsies from the stomach, duodenum, terminal ileum, and colon were obtained based on clinical indications during endoscopic evaluation; therefore, not all segments were assessed in every patient. Gastrointestinal involvement was defined based on available histological data, and patients without biopsy data for a given segment were classified as having no evidence of involvement.

EI was defined as ≥ 15 eos/HPF in esophageal biopsy specimens. Esophageal symptoms were defined as heartburn or dysphagia. Esophageal endoscopic findings were assessed using the EoE Endoscopic Reference Score (EREFS), analyzed as a total score (0–16) and separately for the proximal (0–8) and distal (0–8) esophagus [8].

Continuous variables were compared using the Mann–Whitney *U* test, and categorical variables using Fisher's exact test. Correlations between the EREFS score and esophageal eosinophil counts were assessed using Spearman's rank correlation coefficient. Univariate logistic regression was used to evaluate associations between EI and EREFS scores, as well as gastrointestinal involvement at other sites. Odds ratios (ORs) with 95% confidence intervals (CIs) were calculated. Statistical significance was set at *p* < 0.05.

This original multicenter study was approved by the institutional review boards of the participating institutions [7]. This secondary analysis used de‐identified data, and additional informed consent was waived.

## 3 Results

Among patients included in the original cohort, esophageal biopsy was performed at the discretion of the treating physicians. Twenty‐two patients met the inclusion criteria. Complete biopsy data from all gastrointestinal segments were available for 14 patients. Among the remaining patients, gastric biopsy was not performed in one patient, duodenal biopsy in one, terminal ileal biopsy in three, and both terminal ileal and colonic biopsies in three patients.

EI was identified in eight of 22 patients (36.4%), consistent with previous reports. Clinical characteristics, including demographics, symptoms, PPI use, endoscopic findings, and gastrointestinal involvement, are summarized by EI status (Table 1).

**TABLE 1:** Clinical characteristics of patients by EI status.

	All (*n* = 22)	EI positive (*n* = 8)	EI negative (*n* = 14)	*p* ^a^
Patient characteristics				
Age (median, range, years)	52.5 (24–71)	52.5 (38–71)	54.5 (24–71)	0.83
Male (%)	13 (59.1%)	5 (62.5%)	8 (57.1%)	1.00
Concomitant allergic diseases (%)	17 (77.3%)	6 (75%)	11 (78.6%)	1.00
Atopic dermatitis	5 (22.7%)	2 (25%)	3 (21.4%)	1.00
Allergic rhinitis	7 (31.8%)	3 (37.5%)	4 (28.6%)	1.00
Bronchial asthma	10 (47.6%)	5 (62.5%)	5 (38.5%)	0.38
Urticaria	1 (4.8%)	1 (12.5%)	0 (0%)	0.38
Food allergy	3 (13.6%)	1 (12.5%)	2 (14.3%)	1.00
PPI use (%)	6 (27.2%)	3 (37.5%)	3 (21.4%)	0.62
Symptoms				
Esophageal symptoms (%)	4 (18.2%)	4 (50%)	0 (0%)	**0.0096**
Heartburn	2 (9.1%)	2 (25%)	0 (0%)	0.12
Dysphagia	2 (9.1%)	2 (25%)	0 (0%)	0.12
Abdominal symptoms (%)	22 (100%)	8 (100%)	14 (100%)	—
Nausea/vomiting	11 (50%)	5 (62.5%)	5 (42.8%)	0.65
Dyspepsia	7 (31.8%)	3 (37.5%)	4 (28.6%)	1.00
Appetite loss	8 (36.4%)	4 (50%)	4 (28.6%)	0.38
Abdominal pain	17 (77.3%)	7 (87.5%)	10 (71.4%)	0.61
Abdominal bloating	5 (22.7%)	2 (25%)	3 (21.4%)	1.00
Diarrhea	13 (59.1%)	5 (62.5%)	8 (57.1%)	1.00
Endoscopic findings				
Total EREFS score	0 (0–7)	0 (0–6)	0 (0–7)	0.52
Proximal EREFS score	0 (0–3)	0 (0–3)	0 (0–3)	0.51
Distal EREFS score	0 (0–3)	0.5 (0–3)	0.25 (0–4)	0.28
Total EREFS score ≥ 3 (%)	3 (13.6%)	1 (12.5%)	2 (14.3%)	1.00
Proximal ≥ 3 (%)	2 (9.1%)	1 (12.5%)	1 (7.1%)	1.00
Distal ≥ 3 (%)	2 (9.1%)	1 (12.5%)	1 (7.1%)	1.00
Number of esophageal biopsies	2 (1–4)	2 (1–4)	2 (1–4)	0.39

	All (*n* = 22)	EI positive (*n* = 8)	EI negative (*n* = 14)	OR (95% CI)	***p*** ^a^
GI involvement					
Number of involved GI sites (median, range)	1.5 (1–4)	1 (1–4)	2 (1–4)	0.83 (0.31–1.97)	0.58
Gastric involvement (%)	10 (45.5%)	6 (75%)	4 (28.6%)	7.5 (1.03–54.1)	0.07
Duodenal involvement (%)	10 (45.5%)	3 (45.5%)	7 (50%)	0.6 (0.10–3.53)	0.67
Terminal ileal involvement (%)	11 (50%)	2 (25%)	9 (64.3%)	0.18 (0.02–1.28)	0.18
Colonic involvement (%)	10 (45.5%)	3 (37.5%)	7 (50%)	0.6 (0.10–3.53)	0.67

*Note:* Bold values indicate statistical significance (*p* < 0.05).

Abbreviations: EI, esophageal involvement; EREFS, Eosinophilic Esophagitis Endoscopic Reference Score.

^a^ Comparisons were made between patients with and without EI. Continuous variables were compared using the Mann–Whitney *U* test, and categorical variables were compared using Fisher's exact test. Gastrointestinal involvement was determined based on available histologic data. Patients without biopsy from a given segment were classified as having no evidence of involvement in that segment.

Esophageal symptoms (heartburn or dysphagia) were significantly more frequent in EI‐positive patients (4/8, 50%) than in EI‐negative patients (0/14, 0%; Fisher's exact test, *p* = 0.0096). No patients without EI reported any esophageal symptoms.

The EREFS score was not associated with EI (OR: 1.05 per 1‐point increase; 95% CI: 0.63–1.68; *p* = 0.81). Similar nonsignificant associations were observed for both proximal and distal scores. Furthermore, the number of involved gastrointestinal segments was not associated with EI (OR: 0.83 per additional segment; 95% CI: 0.31–1.97; *p* = 0.68).

Gastric involvement was more frequent in EI‐positive patients than in EI‐negative patients (75% vs. 28.6%), with an OR of 7.5 (95% CI: 1.03–54.1), although this did not reach statistical significance (Fisher's exact test, *p* = 0.07). Terminal ileal involvement tended to be less frequent in EI‐positive patients (OR: 0.18; 95% CI: 0.02–1.28; *p* = 0.18).

Esophageal eosinophil counts tended to be higher in patients with gastric involvement than in those without (median: 25 vs. 2 eos/HPF, *p* = 0.12). No significant correlation was observed between the EREFS score and esophageal eosinophil counts (Spearman's *ρ* = 0.34, *p* = 0.11). In addition, the EREFS score was not associated with the presence of gastric involvement (OR: 1.06 per 1‐point increase; 95% CI: 0.67–2.05; *p* = 0.81).

## 4 Discussion

This secondary analysis of a multicenter cohort provides several insights into EI in non–EoE EGID. First, EI was associated with esophageal symptoms but not with endoscopic severity assessed by the EREFS score, which is known to correlate with eosinophilic inflammation in EoE [8]. This finding suggests that endoscopic findings alone may not reliably predict histologic EI in non–EoE EGIDs.

Second, EI was not associated with the number of gastrointestinal segments involved but tended to be positively associated with gastric involvement (corresponding to EoG as defined by the Pesek criteria). Patients with gastric involvement showed a tendency toward higher esophageal eosinophil counts and more frequent esophageal symptoms, whereas EI appeared to be less frequent in patients with terminal ileal involvement. Although these associations did not reach statistical significance, likely due to the limited sample size, they suggest that EI may reflect an upper gastrointestinal–predominant inflammatory phenotype rather than simply the extent of multisite disease.

Previous studies support this observation. Ketchem et al. reported that EI occurred in approximately 50%–70% of EGID phenotypes with gastric involvement, compared to approximately 30% in those without gastric involvement [2]. Similarly, data from the EGID Partners cohort showed that phenotypes involving the stomach accounted for approximately one‐third of non–EoE EGID cases with EI [3]. In addition, a Japanese nationwide survey reported that EI was present in 26% of EoG and 49% of eosinophilic gastroenteritis cases, but not in EoN or EoC [5]. These findings suggest shared pathogenic mechanisms within the upper gastrointestinal tract, potentially involving gastric acid exposure or alterations in the gut microbiota.

Based on these observations, two possible patterns of EI may exist: one associated with EoE‐like esophageal features (“EoE‐associated EI”), and another associated with gastric involvement (“gastric involvement–associated EI”); however, these interpretations remain hypothetical and require further validation in larger studies.

A previous report suggested that EoE and EI in non–EoE EGIDs share similar molecular mechanisms [9]. In that study, EoE‐like endoscopic findings were observed in 30%–70% of cases in both groups. In contrast, in the present cohort, such findings were observed in only approximately 10% of patients, regardless of EI status. This discrepancy may reflect selection bias, as biopsies in the previous study may have been preferentially obtained from patients with prominent EoE‐like endoscopic abnormalities. In contrast, gastric involvement–associated EI may represent a distinct clinical phenotype characterized by fewer symptoms and less pronounced endoscopic findings [10].

This study had several limitations. Histological evaluation of all gastrointestinal segments was not available for all patients because biopsies were obtained based on clinical indications. The secondary analysis of a previously conducted multicenter cohort study may therefore have led to misclassification of gastrointestinal involvement. However, such misclassification would likely bias the results toward the null rather than generate spurious associations. In addition, the retrospective design and small sample size resulted in wide confidence intervals and limited statistical power, which may have contributed to the lack of statistical significance in several analyses. Furthermore, the relatively small number of esophageal biopsies obtained per patient may have reduced the sensitivity for detecting EI and led to underestimation of its prevalence. Esophageal biopsy was performed at the discretion of the treating physicians, potentially introducing selection bias. Accordingly, these findings should be considered hypothesis‐generating. Prospective studies with systematic biopsy sampling throughout the gastrointestinal segments are needed to confirm these observations. Further, information regarding the effectiveness of PPI therapy was not systematically collected in the original cohort and therefore could not be evaluated in the present study.

## 5 Conclusion

In conclusion, EI in non–EoE EGID appears clinically relevant, particularly regarding its association with esophageal symptoms and gastric involvement, although it does not correlate with endoscopic severity. These findings support the consideration of esophageal biopsies in patients presenting with esophageal symptoms or gastric involvement and may further suggest an upper gastrointestinal–dominant inflammatory phenotype.

## Funding

The authors have nothing to report.

## Consent

This secondary analysis used de‐identified data from the original report, and the requirement for additional informed consent was waived.

## Conflicts of Interest

The authors declare no conflicts of interest.

## Data Availability

Data supporting the findings of this study are available from the corresponding author upon request.
